# Modern History of Cholera Vaccines and the Pivotal Role of icddr,b

**DOI:** 10.1093/infdis/jiab423

**Published:** 2021-08-28

**Authors:** Jan Holmgren

**Affiliations:** University of Gothenburg, Sweden

**Keywords:** cholera vaccine, mucosal immunity, pandemic

## Abstract

The rapid spread of the seventh cholera pandemic over Asia in the 1960s led to several large field studies that revealed that the traditional injectable cholera vaccines had poor efficacy, which led the World Health Organization (WHO) in the 1970s to stop recommending cholera vaccination. At the same time, it stimulated research that has led to the development of the effective orally administered cholera vaccines (OCVs) that today are a cornerstone in WHO’s strategy for *Ending Cholera—A Global Roadmap to 2030*. The first effective OCV, Dukoral, containing a mixture of inactivated *Vibrio cholerae* bacteria and cholera toxin B subunit, was licensed in 1991 and is, together with 2 similar inactivated whole-cell OCVs, Shanchol and Euvichol, currently WHO prequalified and recommended OCVs. This brief review is a personal account of the modern history of the development of these now universally recognized effective tools.

The World Health Organization (WHO) is, from 2010, recommending countries to use cholera vaccination in the public health control of both endemic and epidemic cholera. This recommendation refers to a group of related inactivated oral cholera vaccines (OCVs), which have proved to have consistent and much greater protective effectiveness and acceptability than the old injectable whole-cell vaccines that were abandoned in the 1970s. Three such OCVs—Dukoral, Shanchol, and Euvichol/Euvichol-Plus—are to date recommended and prequalified by the WHO (which means that they can be purchased by United Nations agencies, such as Unicef and GAVI) and additional ones are in the pipeline.

It took a long time before the WHO fully recognized the public health value of OCVs, the first of which, Dukoral, has been available since the early 1990s. The picture has, however, changed completely and the WHO recently called OCVs a game changer in the global fight against cholera. The OCVs are a cornerstone in the global action plan for *Ending Cholera: A Global Roadmap to 2030* [1], launched in 2017 by WHO’s Global Task Force on Cholera Control with the goals to, by 2030, have reduced cholera deaths by at least 90% and eliminated cholera transmission in most of the currently afflicted countries.

It is an honor to have been invited to give this highly personal account of the modern history of cholera vaccines at the 2020 Asian Conference on Diarrhoeal Disease and Nutrition conference hosted by the International Centre for Diarrhoeal Disease Research, Bangladesh (icddr,b) and Bangladesh, which have contributed so much to the cholera vaccine field. My story is focused on the development of inactivated OCVs, especially the ones recommended for public health use by the WHO. Others are better positioned to tell the parallel history of the development of live, attenuated OCVs, which are currently licensed for use in travelers but not yet for public health use.

## FROM PARENTERAL TO ORAL CHOLERA VACCINES

With the rapid spread of the seventh cholera pandemic over Asia in the 1960s, several large field studies were undertaken in East Pakistan (now Bangladesh), India, the Philippines, and Indonesia that revealed that the injectable killed whole-cell cholera vaccines, which had been in wide-spread use for more than 70 years, in fact had very modest efficacy, usually at most 50% for only 3–6 months and limited to adults. Some vaccine preparations had apparently higher efficacy but also gave higher rates of adverse reactions, such as fever and local pain and swelling. This led WHO in the 1970s to stop recommending cholera vaccination.

**Figure 1.**
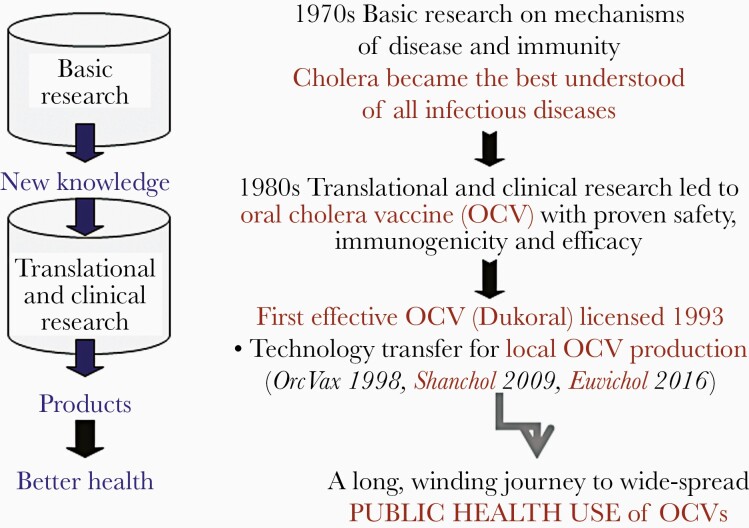
Oral cholera vaccines: from basic research to vaccine development and public health use.

The interest instead rapidly turned to the development of orally administered cholera vaccines. The development of effective OCVs is a good example of basic research being translated into a medical product of benefit for human health (Figure 1). By the early 1970s, the existence of a mucosal immune system, with secretory immunoglobulin A (sIgA) as its main immunoglobulin and activated preferentially by mucosal rather than parenteral immunization, had become established. Simultaneously, in a golden period of international cholera research in the 1970s stimulated by the pandemic, the pathogenesis and immune mechanisms in cholera were clarified to a degree that made cholera probably the best understood infectious disease (for reviews see, eg, [2–5]). For instance, with regard to the mechanisms of disease, we and others defined the A:B_5_ subunit structure and function of cholera toxin (CT) [6, 7]; identify the GM1 ganglioside as receptor for the toxin [8, 9]; and describe the effects of the toxin on intestinal cyclic AMP and fluid transport processes, explaining the often life-threatening diarrhea and fluid loss in patients with severe cholera [10]. Our parallel studies of cholera immunity, reviewed in [4, 5], showed that immune protection was mediated by sIgA antibodies produced locally in the intestine and directed against the bacterial cell wall O antigen lipopolysaccharide (LPS) and CT, and that these antibodies were induced much more efficiently by oral than by parenteral immunization. Protective anti-LPS antibodies were mainly directed against the O1 serogroup-defining epitope A but antibodies against the serotype-specific LPS epitopes B (Ogawa) and C (Inaba) also contributed. Protective antibodies against CT were almost exclusively directed against the CTB pentamer [4]. Maximal, synergistic immune protection was achieved by a combination of anti-CTB and anti-LPS antibodies [4, 11].

These findings provided the basis for the development of the first effective OCV, the combined killed *Vibrio cholerae* O1 whole-cell/cholera toxin B-subunit vaccine (Dukoral), as well as for the subsequent inactivated whole-cell OCVs modeled on this vaccine and OCVs based on live attenuated cholera strains.

## THE DEVELOPMENT OF DUKORAL, THE FIRST EFFECTIVE OCV

Our immunological studies led us to propose, in the late 1970s, that oral immunization with a combined inactivated whole-cell/B-subunit (WC-BS) vaccine should be an effective way to induce protective immunity against cholera [3, 12]. From then on, our cholera vaccine work became increasingly translational. The proposed oral WC-BS vaccine needed to be developed for clinical testing, which also included the need to develop useful immunological methods that would allow measurements of the identified protective mucosal IgA anti-LPS and antitoxin antibody responses in the intestine after immunization in humans and not only, as before antibody responses, in serum.

### Vaccine Production and Quality Control/Quality Assurance Methods

We were collaborating with the Swedish national vaccine producer, the Swedish Bacteriological Laboratory (SBL), which already produced the old injectable cholera vaccine consisting of heat-killed, classical biotype *V. cholerae* O1 Inaba (strain Cairo 48) and Ogawa (Cairo 50) bacteria. We had also, in collaboration with Institut Merieux in France (later Pasteur-Meriex), developed a large-scale method for preparing highly purified CTB from cultures of the hypertoxigenic *V. cholerae* 569B strain, based on a combination of GM1-affinity and gel filtration chromatography [13].

Our first OCV tested in humans therefore consisted of a mixture of SBL’s concentrated injectable vaccine together with chromatographically purified CTB. Soon, however, to also include potential heat-labile protective cell surface antigens, formalin-killed El Tor Inaba (Phil 6973) and formalin-killed classical Ogawa Cairo 50 were added. Later, after we had developed a higher-yield system for recombinant production of CTB (rCTB) [14], the affinity-purified CTB was replaced by rCTB.

New methods for quality control of vaccine component bulks and for quality assurance/release of final vaccine lots were also introduced to complement the bacterial identity, purity, and sterility methods used for the traditional cholera vaccines. Monoclonal antibodies were generated for quantification of LPS by a specific inhibition–enzyme-linked immunosorbent assay (ELISA) method and of CTB by Mancini immunoprecipitation and our earlier-developed GM1-ELISA, as well as for exclusion of any residual toxin-active CT by a CTA-specific GM1-ELISA (and also by a sensitive rabbit skin blueing bioassay).

### Intestinal Lavage-ELISA Methods for Measuring Intestinal-Mucosal Immune Responses in Humans

The intestinal lavage method, developed in collaboration with David Sack at icddr,b, became an almost ideal method to measure local immune responses in the intestine after oral immunization. After fasting, the subject drinks an isotonic solution until a watery diarrhea ensues, and the liquid stool is collected, treated to inhibit protease activity, sterile filtered, and frozen at −70°C until assayed for specific antibody and total immunoglobulin contents by isotype-specific ELISA methods.

Early clinical safety and dose-finding studies in 1979–1982, in first Swedish and then Bangladeshi human volunteers, showed that the WC-BS vaccine was safe and, importantly, that oral immunization in contrast to parenteral immunization effectively induced intestinal IgA anti-CTB and anti-LPS antibody responses as well as an effective IgA immunologic memory to these antigens [15]. The vaccine was given in a bicarbonate buffer to protect the BS component against low stomach pH. Importantly, after 2 oral doses of the WC-BS prototype vaccine the intestinal IgA anti-CTB and anti-LPS responses were fully comparable to those induced naturally in concurrently examined Bangladeshi convalescents from severe clinical cholera [15]. Because such convalescents had been found to have 90% protection against a new clinical cholera episode for the next 3 years, the immune response results suggested that a 2-dose regimen with WC-BS OCV might be equally effective, especially as we decided for the final OCV formulation to double the vaccine dosage to 1 × 10^11^ inactivated bacteria plus 1 mg CTB.

### Phase 1 and Phase 2 Studies and Protection Against Cholera Challenge

Renewed phase 1 and phase 2 clinical studies using the reformulated WC-BS OCV in Swedish, US, and Bangladeshi volunteers confirmed the excellent safety and serologic as well as mucosal immunogenicity. Protection against cholera challenge was tested at the University of Maryland. The WC-BS OCV gave 64% and WC without BS 56% protection against any diarrhea, and both vaccines gave 100% protection against clinically significant cholera [16].

### Phase 3 Field Trials Leading to Licensure of the WC-BS Dukoral Vaccine

At the recommendation of WHO, in 1985 the icddr,b undertook a large placebo-controlled randomized phase 3 trial of WC-BS and WC-only OCVs in Matlab, Bangladesh, led by John Clemens as principal investigator and David Sack as coprincipal investigator. Three oral doses (and by drop-outs, 2 doses or 1 dose) of WC-BS, WC, or *Escherichia coli* K12 placebo were given 4–6 weeks apart to 90 000 women and 2 to 14-year-old girls and boys. The results confirmed the excellent safety and showed that both vaccines conferred significant protection against cholera: protective efficacy (PE) was 85% for WC-BS and 58% for WC OCV over the first 4–6 months, and 50%–55% for both vaccines over 3 years of follow-up (translating to 60%–65% if adult males had been included and equally protected as the adult females). PEs were similar after 2 or 3 doses [17, 18]. Further analyses demonstrated that the WC-BS OCV, but as expected not the WC-only vaccine, also protected against diarrhea caused by enterotoxigenic *E. coli* (ETEC) bacteria producing heat-labile toxin (LT), both strains producing LT only and strains producing both LT and heat-stable toxin (ST), during the first 9 months after vaccination (PE was 67% against all LT^+^ ETEC diarrhea and 86% against severe disease) [19]. In the first year of follow-up, a 48% reduction in admissions for fatal or severely dehydrating diarrhea was seen in the WC-BS group and 33% in the WC group and, as a striking observation, 26% reduction in overall mortality in the WC-BS and 23% in the WC group [20]. The reduction in mortality was limited to vaccinated women, suggesting that sociocultural reasons might have caused a sometimes-fatal barrier for women to seek treatment in time.

A second randomized, placebo-controlled phase 3 trial, now of the current Dukoral WC-BS OCV formulation (with rCTB), was undertaken in military recruits in Peru in 1992. The study subjects were immunologically virgin in that they had not yet been exposed naturally to *V. cholerae* when receiving 2 oral vaccine doses with a 2-week interval; 76% of them were of blood group O, a blood group associated with especially high susceptibility to severe cholera. The results were strikingly similar to those from Bangladesh, confirming the excellent safety of the vaccine and demonstrating 86% PE against cholera during an epidemic 6–8 months after the vaccination [21].

Based on these results, the WC-BS OCV was first licensed in Sweden and other Scandinavian countries in 1991 for use against both cholera and ETEC diarrhea, and from 1993 internationally as Dukoral throughout the European Union and in >40 other countries. It was also prequalified by WHO for purchase through the United Nations system. Large phase 4 effectiveness trials in, for example, Mozambique and Zanzibar have confirmed the excellent safety and demonstrated 80%–90% protective effectiveness after 2 doses of Dukoral against cholera outbreaks occurring 1 or 2 years after vaccination.

## FROM DUKORAL TO ORCVAX/MORCVAX, SHANCHOL, AND EUVICHOL

### Vietnamese OCVs (OrcVax/mOrcVax)

Shortly after the first positive results from the OCV field trial in Bangladesh were published, we were contacted by Professor Dang Duc Trach from Vietnam, who wished Vietnam to produce OCV locally. The WC-only OCV was the primary interest as it would be easier to produce than the full WC-BS vaccine and also could be given without buffer. With SBL’s approval we transferred the WC technology to Vietnam, including the vaccine strains and fermentation, inactivation, and quality control/quality assurance methods.

The first WC OCV produced in Vietnam was from Nah Thrangh; after a few years, however, vaccine production was moved to the newly built Vabiotec production facility in Hanoi. With guidance from John Clemens, the locally produced OCV, given in 2 doses, was tested in 1992–1993 in 67 000 adults and children from age 1 year, in Hue, and proved to be safe and give 66% overall protection against cholera [22]. A few years later, when it was feared that the new *V. cholerae* O139 serogroup might outcompete the O1 serogroup as the main cause of cholera, and a bivalent O1/O139 WC-BS OCV formulation developed in Sweden had been found to be safe and induce intestinal IgA and serum vibriocidal antibody responses also against the O139 component [23], we helped Vietnam to reformulate its OCV to also include formalin-killed O139 strain (the 4260B strain tested in Sweden). To also include the newly discovered toxin-coregulated pilus (TCP) antigen in the vaccine, the formalin-killed classical Cairo 48 strain was replaced with formalin-killed classical 569B bacteria, which expressed high levels of TCP. The reformulated, bivalent O1/O139 OCV was found to be noninferior in safety and O1 vibriocidal antibody immunogenicity when tested side-by-side with Dukoral, as well as to also induce vibriocidal antibodies against *V. cholerae* O139 [24]. The bivalent OCV was licensed for use in Vietnam under trade name OrcVax. The vaccine was also tested in a large placebo-controlled phase 3 trial in Hue, but unexpectedly there were no cholera cases in the study area until 3–5 years after vaccination when cholera (all by the O1 serogroup) reappeared on a major scale, and it was shown that the reformulated OCV used had provided 50% long-term protection [25].

However, it turned out that the use of the hypertoxigenic 569B strain caused vaccine production problems, so occasional vaccine lots had to be discarded due to residual CT. The 569B component was therefore, at our advice, replaced back to the original formalin-killed Cairo 48. Supported by the International Vaccine Institute (IVI; Rodney Carbis and John Clemens) the production processes were also upgraded to comply with international cGMP standards and the reformulated OCV was, in 2009, licensed as mORC-Vax. More than 15 million doses of OrcVax/mOrcVax OCV have been used from 1998 in Vietnam’s national cholera control program, mainly in the Mekong delta in 1998–2006 where cholera at that time was prevalent.

### Shanchol and Euvichol/EuvicholPlus

A problem preventing WHO prequalification and international use of mORC-Vax was that Vietnam’s National Regulatory Agency was not WHO approved. IVI, with the help of Bernard Ivanoff, facilitated a technology transfer from VaBiotech to Shantha Biotechnics in India, which had a WHO-approved National Regulatory Agency. IVI together with the National Institute of Cholera and Enteric Diseases (NICED) in India, also conducted a cluster-randomized, placebo-controlled efficacy trial with the reformulated vaccine in Kolkata, India. Two doses of vaccine provided an overall 65% protective effect over a 5-year follow-up period, although efficacy in children 1–5 years of age was seen for only 2 years [26]. In 2009, the vaccine was licensed in both Vietnam (mORCVAX) and India (Shanchol), and in 2011 Shanchol received WHO prequalification.

Several major cholera outbreaks in 2010, including the devastating epidemic in Haiti with over 8000 deaths in the first year, led to strengthened recommendations from WHO to use OCV for the control of both endemic cholera and cholera outbreaks, and also led to increasing demands for OCV by affected countries. To increase the global OCV supply, IVI helped the South Korean biotech company EuBiologics to set up production of the bivalent OCV. After a first local phase 1 study, EuBiologics conducted a large, randomized phase 2 trial in the Philippines in 2014, showing that its OCV induced vibriocidal responses that were noninferior to those elicited by Shanchol. After also having removed thiomersal from the vaccine, the Euvichol OCV, with the same composition as mOrcVax and Shanchol, obtained WHO prequalification in 2016. Being packaged in more practical plastic vials and renamed, the EuvicholPlus OCV is now the main vaccine used in the global OCV stockpile [27]. With increasing demand and purchase from GAVI, annual production capacity of EuvicholPlus now exceeds 25 million doses and will soon exceed 50 million doses.

## HERD PROTECTION—A KEY FINDING FOR PUBLIC HEALTH USE OF OCVS

A key finding for the public health expanded use of OCVs is that, in addition to their specific vaccine efficacy, they confer strong indirect, so-called herd protection in vaccinated communities. This was first shown by Ali et al [28] and has been repeatedly confirmed, for example, in studies of Dukoral in Zanzibar and of Shanchol in India and Bangladesh [29, 30]. The herd protection is due to the ability of OCV to reduce person-to-person transmission of cholera, thus providing protection also in nonvaccinees who reside in vaccinated neighborhoods as well as enhanced protection in vaccinees; its magnitude is proportional to the vaccination coverage of the target population. Already from 50% or higher coverage the available OCVs can result in virtually complete elimination of cholera.

Herd protection can markedly increase the overall protective impact of OCVs in vaccinated communities. This makes OCVs very cost effective by WHO measures with regard to both lives saved and disease averted in relation to expense, which no doubt was important for the rapid change in the public health perception of OCVs.

## THE GLOBAL OCV STOCKPILE

The event that was pivotal for a rapid change in attitude to public health use of OCV was the cholera epidemic in Haiti in 2010. It not only led to the strengthened recommendations from the WHO to use OCVs for the prevention and control of both epidemic and endemic cholera but also to the important decision to establish, with support from GAVI, a global OCV stockpile. The stockpile, which was started in 2013 with only 2 million doses for use primarily in cholera outbreaks, has now with financial support from GAVI increased to 20–25 million doses annually. More than 50 million doses have been used to date in more than 100 mass vaccination campaigns in 22 countries. The focus is increasingly on preventive vaccination in cholera hotspots in Africa and Asia in accordance with the Global Roadmap strategy.

## NATIONALLY LICENSED BUT NOT WHO-PREQUALIFIED OCVS

Beside the 3 currently WHO-prequalified OCVs, several other OCVs have received licensure in 1 or more countries but are not WHO prequalified (Table 1). A number of additional future OCVs are in different stages of development (Table 2). Both of these categories of OCVs are further described in, for example, [5, 31, 32].

**Table 1.** Nationally Licensed but Not World Health Organization Prequalified OCVs

Vaccine (Producer, Country)	Type of Vaccine	Licensure Countries	Reference
mOraVax (VaBiotech, Vietnam)	Inactivated O1/O139 whole cells (model vaccine for Shanchol, Euvchol, and Cholvax)	Vietnam	[31,32]
Cholvax (Incepta, Bangladesh)	Inactivated O1/O139 whole cells	Bangladesh	[31,32]
OraVacs (Shanghai United Cell Biotechnology, China)	Enteric-coated capsule vaccine modeled on Dukoral’s composition of inactivated O1 whole cells + rCTB	China and Philippines (for protection against cholera and ETEC diarrhea)	[31,32]
Vaxchora (PaxVax, United States)	Live, attenuated OCV containing lyophilized *Vibrio cholerae* CVD 103-HgR classical biotypeO1 Inaba bacteria (derivative of 569B)	United States and European Union as traveler’s vaccine against cholera	[31–33]

Abbreviations: ETEC, enterotoxigenic *Escherichia coli;* OCV, oral cholera vaccine; rCTB, recombinant cholera toxin B.

**Table 2.** New OCVs Under Development

Type of OCV	Description	Development Stage
Simplified liquid compositions of current OCVs	Formalin-killed Cairo 50 (Classical/Ogawa) and Phil6973 (El Tor/Inaba); developed in South Korea	Preclinical development in South Korea
	Hillchol, formalin-killed Hikojima El Tor strain MS1568; developed in India and Sweden	Planned phase 3 testing in India
	Isochol, formalin-killed, cocultured isogenic El Tor Ogawa and Inaba strains; developed in Sweden	Preclinical development in Sweden
Thermostable dry formulation capsule OCV	DuoChol, enteroprotected dry formulation capsule OCV containing a lyophilized mixture of formalin-killed cocultured isogenic El Tor Ogawa and Inaba strains and rCTB; developed in Sweden	Preclinical development in Sweden
Live attenuated OCVs	Genetically engineered *Vibrio cholerae* O1 strains with deletions of *ctx* and other mutations:	
	• Peru 15, derived from an O1 El Tor Inaba clinical isolate from 1991 in Peru; developed in United States	Completed phase 1 (including challenge) in United States and phase 2 immunogenicity testing in Bangladesh
	• El Tor Ogawa strain 638; developed in Cuba	Completed phase 1 (including challenge) in USA and phase 2 immunogenicity testing in Cuba
	• VA 1.4 El Tor Inaba; developed in India	Completed phase 1 immunogenicity testing in India
	• IEM 108 El Tor Ogawa; developed in China	Completed phase 1 immunogenicity testing in China
	• HaitiV, derived from a variant El Tor O1 Ogawa isolated in Haiti; developed in United States	Preclinical development in United States

Adapted from Holmgren 2021 [5] to which the reader is referred for further details and literature references.

Abbreviations: OCV, oral cholera vaccine; rCTB, recombinant cholera toxin B.

## PIVOTAL ROLE OF icddr,b IN THE DEVELOPMENT AND USE OF OCVS

The icddr,b has played a major scientific role in the modern history of cholera vaccines. For instance, as referred to above, it was scientists from icddr,b who, often in fruitful collaboration with international scientists:

1. conducted many of the large field trials in the 1960s that eliminated the injectable cholera vaccines;
2. showed that convalescents from natural cholera disease had a 90% reduced risk of having a second cholera episode for several years;
3. introduced the intestinal lavage and other pivotal methods for studying intestinal immune responses to cholera infection and immunization in humans;
4. engaged in the clinical studies guiding the composition of the first effective OCV (Dukoral) and undertook the large pivotal field trial leading to its international licensure;
5. identified the strong herd protection induced by OCVs and its important impact on the overall effectiveness of OCVs;
6. prevented an otherwise almost certain outbreak of cholera among Rohingya refugees to Bangladesh in 2017 by providing rapid immunization with OCV;
7. have promoted and supported local OCV manufacturing in Bangladesh and been a key partner in the clinical development of recent OCVs, such as Cholvax, to-be-licensed Hillchol, and yet others in the pipeline; and
8. of major importance, have provided advice and support to the host country Bangladesh for it to become the strongest and best-informed advocate and role model on the global scene for use of OCV in the control of cholera in afflicted countries.

Countless scientists from the icddr,b have played major roles in cholera vaccine research. John Clemens and Firdausi Qadri stand out as scientific giants in the cholera vaccine field and have had a pivotal role in the clinical development, evaluation, and global introduction and advocacy of OCVs. If I add to them, in chronological order of my own collaboration with them, John Craig, Henry Mosley, Michael Merson, Roger Glass, Robert Black, David Sack, M. Yunus, and M. Ali as just a few, it illustrates how many brilliant scientists who over more than 5 decades have made icddr,b a mecca for cholera vaccine research.

## Notes

***Acknowledgments*.** I thank all of my many collaborators through the years in Sweden and internationally. In particular, I wish to acknowledge the invaluable contributions of Ann-Mari Svennerholm as joint developer of the Dukoral vaccine and for continued collaboration and support over the years; Marianne Jertborn for her important role in the early clinical development of both Dukoral and the first bivalent OCV; the late Dung Duc Trach at NIHE, Vietnam for his pioneering work on local production of OCV in Vietnam; John Clemens, David Sack, and Firdausi Qadri at icddr,b for their pivotal roles since the 1980s in the clinical development, evaluation, and advocacy of OCV and for continued collaboration and friendship; Bernard Ivanoff at WHO, Roger Glass at NIH, Lennart Freij at Sida/Sarec, and Robert Hall at NIH for collaboration, support, and friendship; Marianne Lindblad, Gudrun Wiklund, Joaquin Sanchez, Cecil Czerkinsky, Michael Lebens, and Stefan Karlsson-Nordquist and many others at University of Gothenburg for long and good collaboration; SBL, icddr,b, and Hilleman Labs for valuable collaboration; and the Swedish Research Council, Swedish Sarec-Sida, the KAW and MMW Wallenberg Foundations, the BMGF, the Wellcome Trust and others for financially supporting our work through the years.

***Potential conflicts of interest.*** The author has been engaged in the development, testing, and advocacy of many of the cholera vaccines described in the article, mainly Dukoral but also OrcVax/mOrcVax, Shanchol, Hillchol, Isochol and Duochol, and he is a co-inventor on patent applications relating to the three latter vaccines. The author has submitted the ICMJE Form for Disclosure of Potential Conflicts of Interest. Conflicts that the editors consider relevant to the content of the manuscript have been disclosed.
